# Supplementary material for: Influenza A Virus NS1 Protein Structural Flexibility Analysis According to Its Structural Polymorphism Using Computational Approaches
Source: Int J Mol Sci. 2022 Feb 4;23(3):1805. doi: 10.3390/ijms23031805 (PMC8836794; doi:10.3390/ijms23031805)
Supplement: Supplementary file 1 [file ijms-23-01805-s001.zip › ijms-1561155-supplementary.pdf]

International Journal of
Molecular Sciences

MDPI

Supplementary Materials:

The following are available online at https://www.mdpi.com/article/10.3390/ijms23031805/s1.

Figure S1:

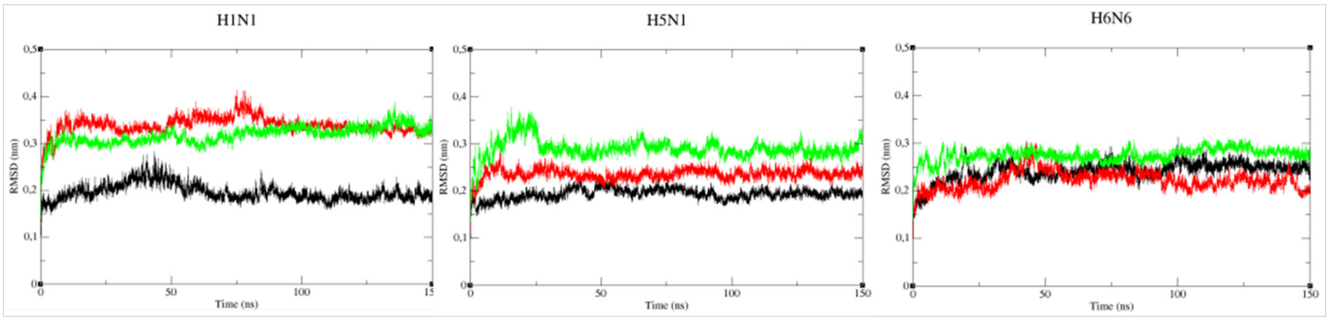

Figure S1. RMSD curves of the RBD domain for the three strains in the three forms (closed in black, semi-open in red and open in green).

Figure S2:

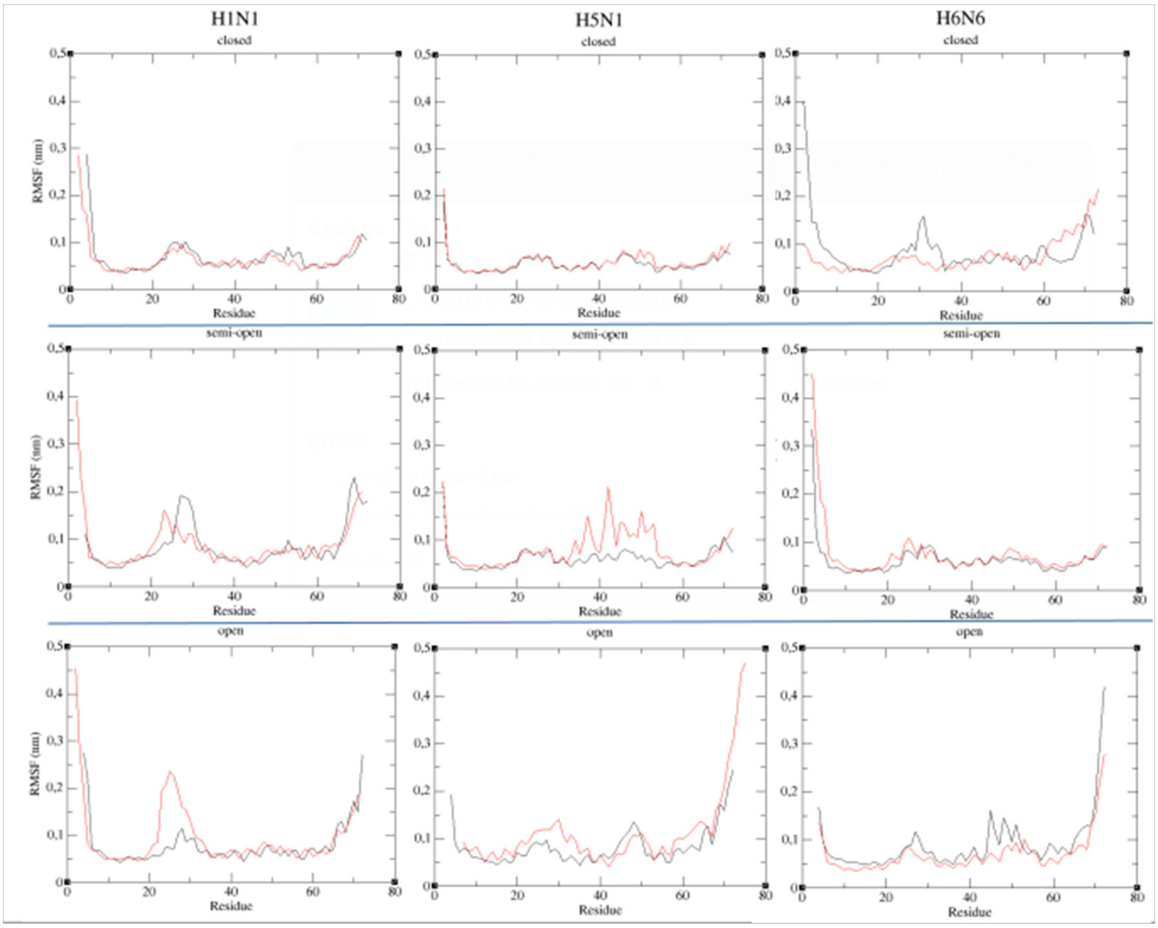

Figure S2. (Cα RMSF) curves of the two chains of the RBD domain (residues 1 to 72) for the three forms. The chains A and B colored in black and red respectively.

Figure S3:

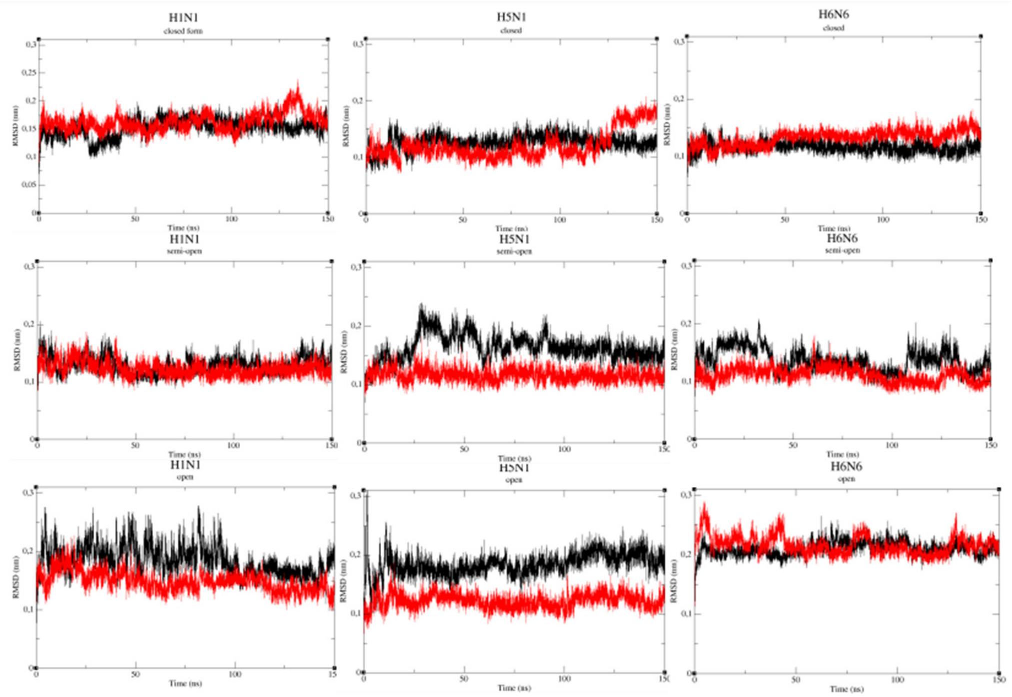

Figure S3. (Cα RMSD) curves of each ED monomers A (colored in black) and B (colored in red) fittest on themselves for the three strains and the three forms.

Figure S4:

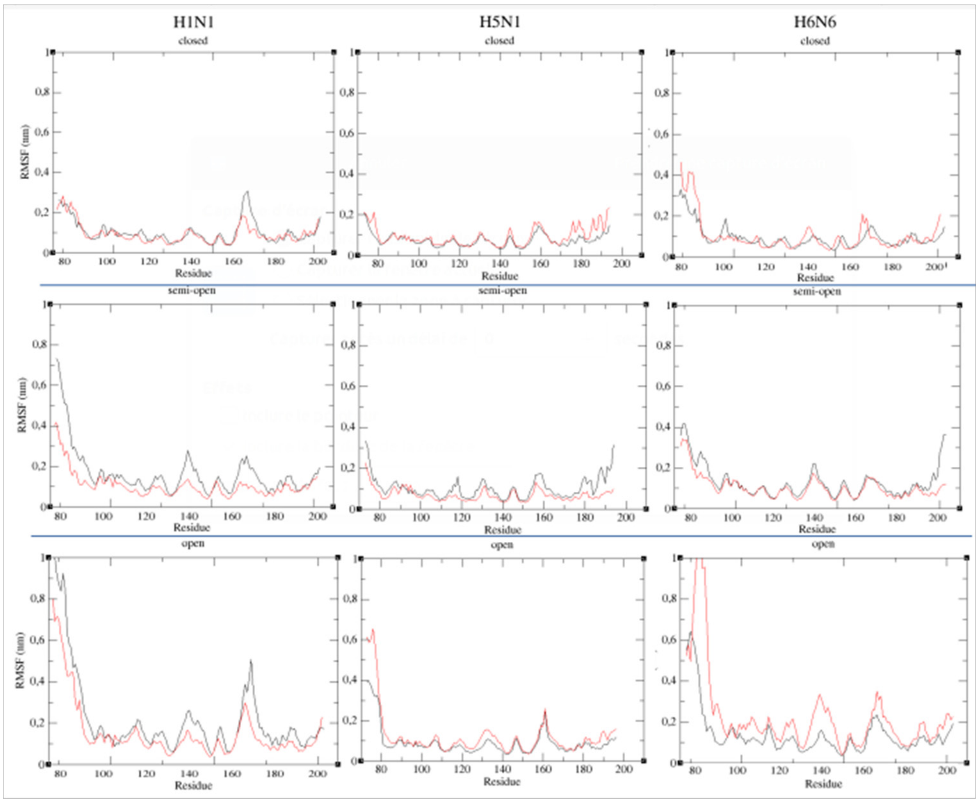

Figure S4. (CαRMSF) curves of the two chains of ED domain including the linker region for the three strains in the three forms. The chains A and B colored in black and red respectively.

Figure S5:

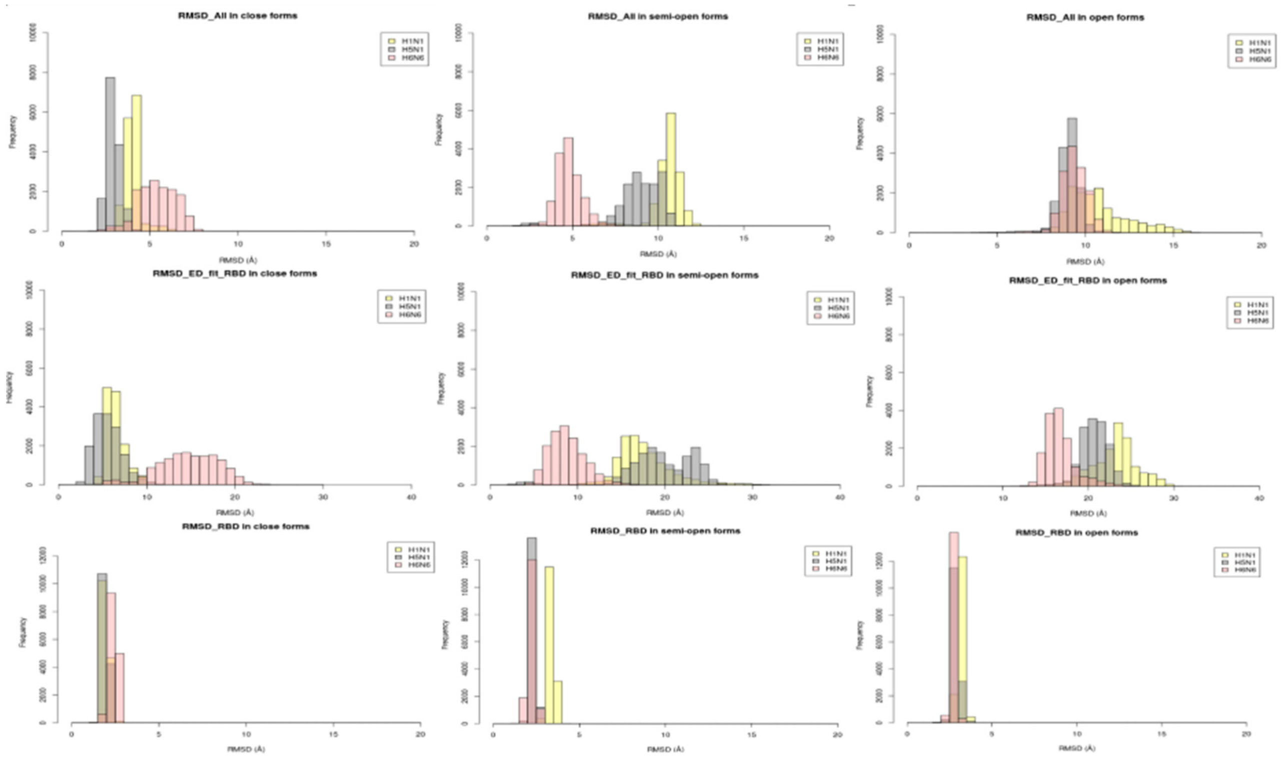

(**a**)

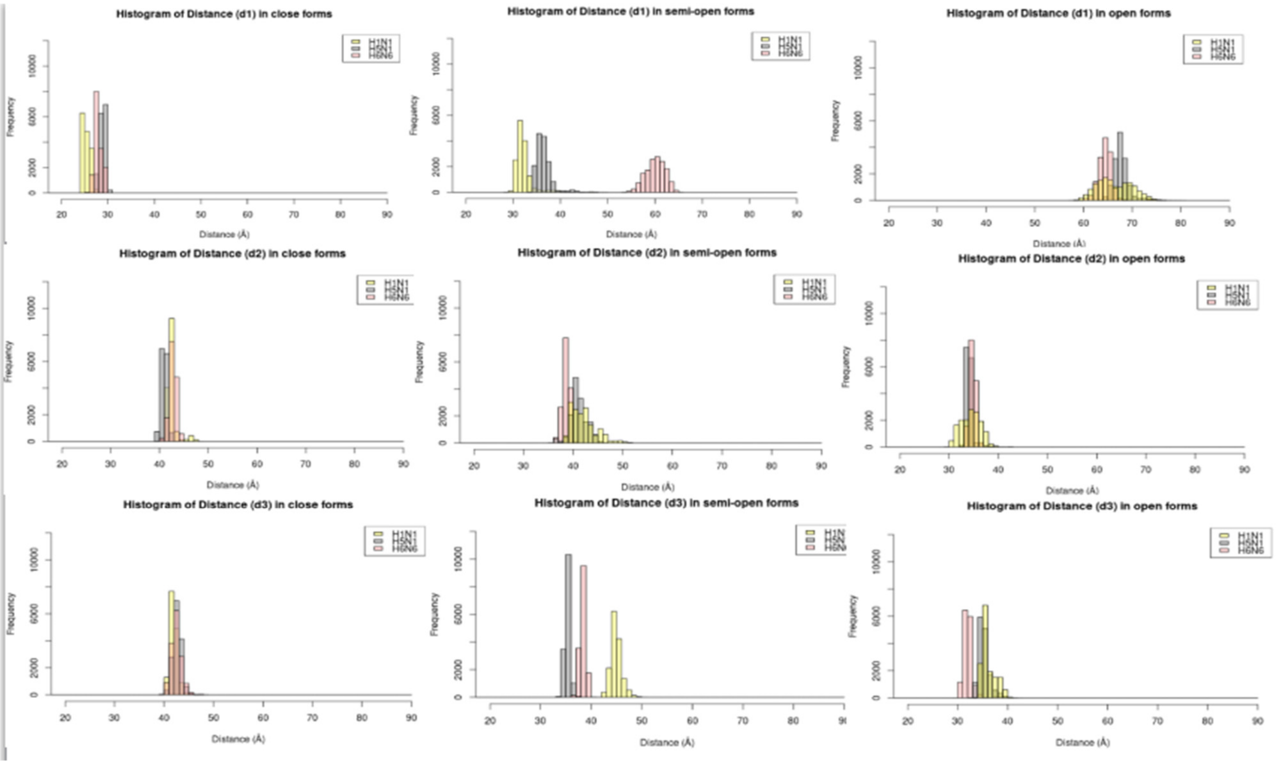

(**b**)

Figure S5: (a) Histograms of RMSD values for the different strains (H1N1 in yellow, H5N1 in grey and H6N6 in pink): RMSDs are calculated on the whole protein, on the ED after fitted on RBD and on RBD respectively. Histograms are represented respectively for NS1 in the closed form (on the left), in the semi-open form (in the middle) and in the open form (on the right). (b) Histograms of the three distance values for the different strains (H1N1 in yellow, H5N1 in grey and H6N6 in pink): distances are calculated between the ED domains (d1), between the RBD dimer and the ED A chain (d1) and

between the RBD dimer and the ED B chain (d3) respectively. Histograms are represented respectively for NS1 in the closed form (on the left), in the semi-open form (in the middle) and in the open form (on the right).

Table S1:

**Distance (Å)**	**Closed form**			**Semi-open form**			**Open form**		
**Strains**	**H1N1^{HM}**	**H5N1^{HM}**	**H6N6^{HM}**	**H1N1^{XR}**	**H5N1^{HM}**	**H6N6^{XR}**	**H1N1^{HM}**	**H5N1^{XR}**	**H6N6^{HM}**
**Distance d1**	27.7	28.1	27.8	47.0	43.3	59.8	74.4	68.9	75.3
**Distance d2**	40.2	39.9	39.6	49.5	35.6	37.9	37	34.2	39
**Distance d3**	42.9	42.4	42.6	46.6	33.7	38	37.5	34.7	36.6

Table S1: Table of distances between the different domains of NS1 for three strains in the three forms on the initial structures. The distance (d1) corresponds to the distance between the geometric center of the two ED monomers. The distances (d2, d3) correspond respectively to the distance between each of the geometric center of the monomer chains A and B of the ED domain and the geometric center of the RBD dimer.

Table S2:

**Strains/ Forms**	**Closed form**		**Semi-open form**		**Open form**	
**strains**	**H6N6^{HM}**		**H6N6^{XR}**		**H6N6^{HM}**	
**RMSD values (in Å)**						
**Time of simulation (in ns)**	150	340 (extended)	150	298 (extended)	150	180 (extended)
**RMSD All**	5.0 ± 1.1	6.5 ± 1.3	4.2 ± 0.7	4.7 ± 0.7	8.9 ± 7.9	9.3 ± 0.7
**RMSD RBD**	2.4 ± 0.2	2.3 ± 0.1	2.2 ± 0.2	2.1 ± 0.1	2.7± 0.1	2.7 ± 0.1
**RMSD ED fit to RBD**	14.7 ± 3.4	17.5 ± 4.1	8.8 ± 2.0	8.8 ± 0.2	16.6 ± 1.8	16.6 ± 1.7

Table S2: Average Cα RMSD for extended simulations of H6N6 strain in the three forms (closed, semi-open, open) during the trajectory. RMSD_All is the average RMSD calculated over the whole protein, RMSD_RBD is the RMSD of the RBD dimer after fitting to the RBD, RMSD ED_A and ED_B are the RMSD of each monomer A and B of the ED domain calculated independently and RMSD ED fit on RBD corresponds to the RMSD of the ED domains fitting to the RBD dimer.

Figure S6:

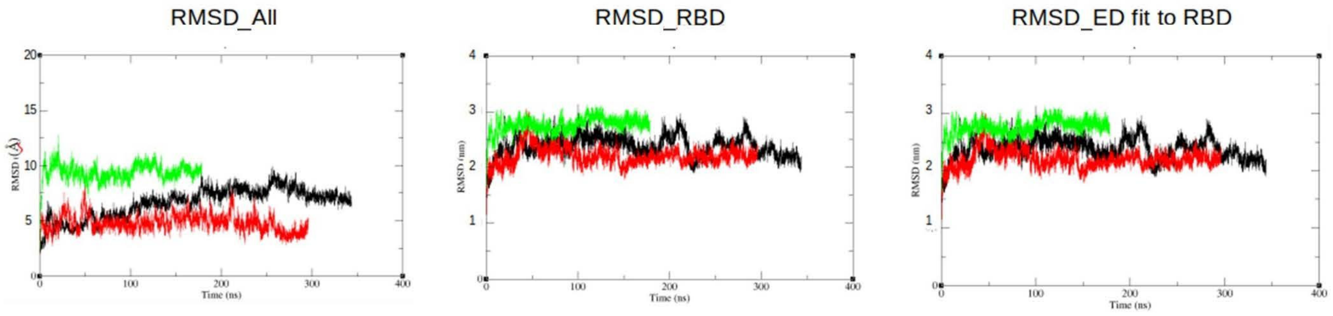

Figure S6. Cα RMSD for extended simulations of H6N6 strain in the three forms (closed, semi-open, open) during 340ns, 298ns and 180ns respectively. RMSD_All is the average RMSD calculated over the whole protein, RMSD_RBD is the RMSD of the RBD dimer after fitting to the RBD, RMSD ED_A and ED_B are the RMSD of each monomer A and B of the ED domain calculated independently and RMSD ED fit on RBD corresponds to the RMSD of the ED domains fitting to the RBD dimer. .
